# Traditional and Domestic Cooking Dramatically Reduce Estrogenic Isoflavones in Soy Foods

**DOI:** 10.3390/foods13070999

**Published:** 2024-03-25

**Authors:** Souad Bensaada, Gabriele Peruzzi, Laurent Cubizolles, Muriel Denayrolles, Catherine Bennetau-Pelissero

**Affiliations:** 1 Univ. Bordeaux, CNRS, INSERM, ARNA, UMR 5320, U1212, F-33000 Bordeaux, France; souad.bensaada@u-bordeaux.fr; 2 Berkem, Rue Jean Duvert, 33290 Blanquefort, France; gabriele.peruzzi@biopress.fr (G.P.); laurent.cubizolles@berkem.com (L.C.); 3 Feed & Food Department, Bordeaux Sciences Agro, 33175 Gradignan, France; muriel.denayrolles@agro-bordeaux.fr; 4 CBMN, UMR CNRS 5248, 33607 Pessac, France

**Keywords:** soy food, phytoestrogens, genistein, daidzein, cooking practices, water treatments, human exposure, health

## Abstract

Soybean is a pulse which has considerable nutritional value due to its high protein, fibers and polyunsaturated fatty acid (PUFA) contents. It also contains phytoestrogenic compounds that definitely hinder its recommendation for general consumption. Contrary to ancient times, when soybeans were boiled, modern commercial soy foods can contain up to 150 mg/100g of estrogenic isoflavones. Interestingly, current estimations of isoflavone intake in the literature do not distinguish between the origins of soy food, i.e., whether it is homemade or commercial. As a result, the isoflavone exposure in Asian countries may well be overestimated. This study aims to demonstrate, based on step-by-step monitoring of isoflavones, that traditional and domestic treatments, leveraging isoflavones water-solubility, can indeed significantly reduce their content in soy foods. Indeed, when compared to commercial foods, the isoflavone content was found to be 20, 2.6, 4.5 and 9.8 times lower in “homemade” soy juice, tofu, tempeh and miso, respectively. Additionally, water soaking was found to reduce the isoflavones levels in soy-textured proteins by more than 70%. Hence, this simple process has the potential to help drastically reduce overall xenoestrogens exposure. This study could serve as a basis for establishing the isoflavones Reference Dose and issuing food safety guidelines.

## 1. Introduction

Soybean (*Glycine max* (L.) Merr.) is recognized as the foodstuff with the highest concentration of isoflavones (IFs), which exhibit estrogenic activities. Compared to other xenoestrogens, IFs bear the closest resemblance to estradiol and are highly concentrated in food sources [1,2]. The effects of IFs can vary from beneficial to adverse, contingent on the physiological status of the consumer and the ingested quantity [3]. In 2008, the National Toxicology Program (NTP) of the USA classified genistein (GEN), the primary soy-isoflavone, as reprotoxic and carcinotoxic in rats at a dosage of 35 mg/kg/day [4,5]. Given the absence of a reliable NOAEL (No Observable Adverse Effect Level), Food Safety Agencies should propose a common Reference Dose for the estrogenic IFs that should not be exceeded. This limit should also consider the actual exposure in Asian countries. Estimating this exposure necessitates reliable measurements in foodstuffs, an issue this study aims to address.

Due to potential adverse effects in certain population categories, it may be beneficial to reduce the levels of estrogenic IFs in the diet intended for the general population, reserving IFs for specific applications and circumstances. This cautionary approach is often disputed, with arguments that soy is traditional in Asia and that no adverse effects, or even beneficial ones, have been reported in the past [6]. However, does high soy consumption necessarily equate to high IFs exposure? 

A close examination of soy’s history in China reveals that written testimonies were frequently lost and reconstructed over the centuries, making it challenging to trace. The first reliable reports date from the Han dynasty (206-BCE to 220 CE). They described agricultural practices that could be dated back 2000 years before reporting, making it difficult to distinguish factual information from myths [7]. Although soy has been cultivated for ages, the destruction of written sources prevents the confirmation of a time period for soybean’s use as a foodstuff. For instance, it was primarily used as a green manure before the Han dynasty, when the major crops cultivated were millet, wheat, rice, hemp, mung beans, and sesame [8]. In fact, soybean can be cultivated even in low-quality soils due to its ability to fix nitrogen in soils, enhancing subsequent cultures. As such, it was used for over 1000 years in quite complex crop rotations [9]. Soy was easily stored for up to three years, since it contains many antinutritional factors which reduced pest (rodents or birds) attacks in the fields or during seed storage. Initially, it was mainly used to feed animals and was considered a starvation and war crop around 100 BCE [7,10]. It was not highly appreciated due to its poor taste and the flatulencies and the stomach pains it induced. The most effective method to improve soybeans’ digestibility at that time was to boil them. Early fermented soy foods were seemingly reported in 1200 BCE in Korea. Soy was then considered a potential food for human beings [9]. In China, according to Baraibar-Norberg and Deutsh [7], *shi* (fermented soybeans) was found in Han tombs dated from 200 to 150 BCE. Fermentation, which allowed better conservation and enhanced taste, required the use of clean and sterile beans before the development of specific molds, implying water boiling [9]. Soy curd like tofu seemed to appear during the late Han dynasty. Again, the traditional recipes included several cleanings and rinses as well as prolonged water boiling [11].

Such treatments applied to beans or dehulled beans have been reported to yield the inactivation of antinutritional factors and the removal of estrogenic IFs while preserving proteins [12], fibers, and polyunsaturated fatty acids (PUFAs) among ω3 and ω6 families [13]. In contrast, modern industrial processes primarily involve physical constraint treatments such as pressing and extrusion, which may lead to some concentration of IFs. Therefore, when dietary reports in the literature suggest that Asian populations are exposed to high levels of IFs, based solely on high soy food consumption, this appears to be a questionable assumption if the food origin is not specified. 

Additionally, when data associating estimated IFs intakes and IFs in body fluids are gathered, a discrepancy emerges between Western and Asian populations. The same estimated exposure leads to 1 to 1.5 µM in Western people’ blood, while it is about 0.2 to 0.3 µM for Asians. This was previously questioned by Vergne et al., in [14], when the pharmacokinetics of GEN and Daidzein (DAI) were assessed concurrently in French and Chinese students. The study revealed only a small difference in the C*_max_* of DAI during chronic soybean challenge and it was hypothesized that, in Asian students, the gut flora may negatively influence the IFs bioavailability. However, this finding alone could not explain the significant difference observed in Asians and Westerners’ body fluids concentrations of IFs. Thus, the hypothesis of a misestimation of IFs intakes remained.

This study was designed to address this issue. Its objective is to demonstrate that traditional recipes, and by extension the domestic ones inspired by them, reduce significantly IFs levels in Asian soy foodstuffs. Hence, GEN and DAI concentrations were measured on soy foods prepared under domestic conditions by ELISA. As the exact step for potential IFs removal was unknown, the processes were analyzed step by step to determine their impacts on the concentration of these two major estrogenic IFs. The results should indicate that traditional exposure to IFs in Asia is not as high as suggested by recent data [15,16] which established the IFs exposure at a median of 13.5 mg/day in China and at 21.4 mg/day in Japan.

The foodstuffs selected were tempeh and miso for fermented food and soy juice and tofu for unfermented food, these being among the most common traditional dishes. Soy textured proteins were also treated under domestic conditions since they can be used by Western consumers. A comparison was then made with commercial industrial equivalent products analyzed here and in a previous study by the same assay technique. The health effects of estrogenic IFs are then discussed as well as the most probable consequences of this finding on the estimation of Asians’ IFs exposure.

## 2. Materials and Methods

### 2.1. Materials

All soy matters, i.e., whole and dehulled soybeans and textured soy proteins, were supplied by the organic producer of soy proteins Biopress^®^/Berkem^®^ (Tonneins, France). Raw matters were acquired from a local organic farmers’ union: Bioprogress (Fourcès, Gers, France), which follows Demeter specifications. The soybeans farms were located in the Lot et Garonne and Gers departments. Both whole and dehulled soybeans were obtained from the ISIDOR cultivar, but from two different storage silos. Dehulled soybeans were sifted before use to remove soybean dust. Textured soy proteins were sorted in four different particle-size groups: large (12–20 mm), medium (8–12 mm), fine (5–8 mm) and extra-fine (2.5–5 mm). Rice-koji ferments, i.e., *Rhizopus oligosporus* for tempeh and *Aspergillus flavus* var *orizae* for miso, as well as miso starter were bought from an organic store (Biocoop, Bègles, France). The plain tofu and tempeh used in the rinsing experiment also came from the same local organic store. They both came from the “Soy” brand. The tofu was composed of water, 25.9% dehulled soybeans, gellant (calcium sulphate), and nigari. The tempeh was composed of 99.3 % dehulled soybeans precooked in water, cider vinegar and ferments. The commercial products analyzed as a basis for comparison given in Table 1 were purchased during the last 5 years from French supermarkets located around Bordeaux.

The chemical reagents were acquired from MERK (Fontenay-sous-bois, France) and Sigma-Aldrich (Saint Quentin Fallavier, France), unless otherwise stated. The samples and reagents aqueous solutions were made in ultrapure water obtained from an Elga Veolia^®^ (High Wycombe, UK) instrument. 

### 2.2. Methods

#### 2.2.1. Traditional Recipes

##### Soy Juice

Soy juices were prepared from either 100 g of dehulled beans or whole beans. The first soaking step in 1L of water lasted 12 h, during which the water was renewed 0, 1, 2, 3 or 4 times. Then, the beans were mashed in 700 mL of either soaking water or clean water. The different mixtures obtained were individually filtered through a piece of fabric and the corresponding okara kept aside. For each type of juice collected, 350 mL was diluted twice with water to reach the commercial concentration of soymilk. After a boiling step to reduce all antinutritional factors, it was allowed to cool down to room temperature before storage at 4 °C or −32 °C. At each stage of the process, three samples were collected and analyzed individually.

##### Tofu

Tofu samples were prepared from the different soy juices obtained just before the last dilution step as previously described. After boiling, nigari (MgCl_2_) was added at a final concentration of 5 g/L and the juice was left to curdle for 30 min. The curds were poured onto a tofu press and the whey discarded by pressing for 30 min at 25,500 Pa.

##### Tempeh

The recipe is based on a traditional process described by Fernandez-Lopez et al. [17]. Dehulled beans were rinsed 3 times with tap water before being cooked in boiling water for 20 min and then left to soak in cooking water for an extra 20 min. During this first cooking step, the foam that formed on top of the water was removed. These precooked beans in turn underwent the same 3 processing stages as the dehulled beans. After the second cooking, beans were lightly dried and spread on a baking sheet in an oven at 60 °C for 10 min. Finally, the beans were mixed with white vinegar and *Rhizopus oligosporus* ferment, reaching concentrations of 6 mL/100 g and 500 mg/100 g, respectively. The mixture was poured in zip-lock plastic bags, which were pierced with a fork and left in a heat chamber at 28 °C for 72 h.

##### Miso

The traditional recipe for miso was taken from The Japanese Lab [18], which describes how to make light-colored miso. First, 100 g of dehulled beans were cooked in boiling water for 3 h, during which the boiling water was renewed twice. They were cooled down to room temperature and mixed with the equivalent amount of rice-koji, i.e., rice pre-incubated with *Aspergillus flavus* var *oryzae* and 133 mg of miso starter. The obtained mixture was formed into balls which were deposited and pressed into a large-necked glass jar to avoid air pockets. Its surface was sprinkled with salt and then covered with a food-grade plastic film supporting a ballast. The jar was closed and the mixture was left to ferment for 8 months.

##### Hydrated Soy Proteins

Four sizes of textured soy-proteins were tested. In each case, 100 g of matter was soaked in 1 L of tap water to hydrate for 30 min, during which 0, 1, 2 or 3 water renewals were performed. In the case of 1 renewal, it occurred after 30 min; for 2 renewals, they occurred after 15 and 30 min; for 3 renewals, they occurred every 10 min. Two water temperatures were tested: 22 °C and 65 °C.

##### Commercial Tofu and Tempeh

A consumer portion, either 100 g of tempeh or 125 g of tofu, was immersed in 1 L of deionized water for a total of 15 min, with two water changes at 5 and 10 min, before which the foodstuff was dried on absorbent paper and weighted. Water was gently stirred for 1 min at each water renewal. Samples were collected at 0, 5, 10 and 15 min of soaking. IFs, i.e., GEN and DAI in aglycone forms, were assayed at each time set.

#### 2.2.2. ELISA

Despite recommendations from the French Food Safety Agency in 2005 [19], declaration of isoflavones content in soy-based foodstuffs is still not mandatory in France. However, considering the functional value of these substances, ELISAs were developed by our team in the late 1990s and have since been used to analyze these polyphenols in foods, biological fluids [20], and recently in hair [21]. This technique was validated in an international ring test monitored by TNO (Nederlandse Organisatie voor Toegepast Natuurwetenschappelijk Onderzoek, The Netherland) and results can be found in the work of Bennetau-Pelissero et al. [20]. Although this ring test showed considerable heterogeneity in isoflavones determination between laboratories, this was essentially due to inadequate extraction procedures in some cases. Nevertheless, data obtained using ELISA appeared reliable and comparable to the best chromatographic techniques challenged in this trial when aglycone forms were considered. Additionally, ELISAs have been validated in two studies designed to test food-frequency questionnaires assessing recent [22] and usual soy intakes [21]. To date, there is no legal technique for isoflavones determination in France.

##### Samples Preparation

To perform the IFs analyses via ELISA, the samples were first subjected to an extraction as described below. Thereby, 1 g of mortar-crushed soy-based material was dispersed into 50 mL of water by a two-step stirring at room temperature for 20 min and then at 100 °C for 10 min. After cooling down to room temperature, 500 µL samples were collected in triplicate under stirring to ensure homogeneity. Two milliliters of acetate buffer (sodium acetate 0.1 M; EDTA 0.14 M; 100 UI.mL^−1^ penicillin G (Sigma, P-3032); 0.1 mg.mL^−1^ streptomycin (Sigma, S-6501)) at pH 5 were added to each sample vial together with 10 µL of β-glucuronidase aryl-sulfatase from *Helix pomatia* (Roche^®^, 10127698001, Mannheim, Germany) to allow the digestion of glycosylated IFs. The samples were then incubated overnight at 37 °C with shaking [23]. Afterwards, the extraction of aglycone compounds was performed: 3 mL of acidified ethyl-acetate (500 µL HCl 38% per L) was added, and the vial vortexed for 30 s, centrifuged at 500 g (Jouan^tm^ CR3, Fisher Scientific, Illkirch, France) for 10 min at 4 °C and finally stored at −22 °C to allow phase separation. The organic phase containing the IFs in aglycone forms was evaporated to dryness using a Speed-Vac (Thermo-Electron^tm^ Corporation, Fisher Scientific, Bordeaux, France). Then, each sample was diluted in 0.5 mL of assay buffer, i.e., phosphate-buffered saline (PBS) 0.01 M, 0.9% NaCl, 0.2% Tween, 1% DMSO, pH 7.3, and sonicated when required. Samples were stored at −32 °C until processed for IFs analysis. 

To assess the digestion and extraction recovery, the hydrolysis by β-glucuronidase aryl-sulfatase was monitored using genistin (EXTRASYNTHESE™, 1325 S, Genay, France), a pure compound used as a control reagent in an external standard run in parallel to each measured sample. The compound was dissolved at 1 mg.mL^−1^ in DMSO as the stock solution. In these operating conditions, the hydrolysis performance was always between 87 and 103%. In addition, a sample of chocolate soymilk was run similarly to the tested samples. It was considered to be the plate control. Recoveries greater than 100% can be explained by the high accuracy of ELISA measurements and inter- and intra-assay variations.

##### Assay

GEN and DAI were assayed in soy matter and treatment water using ELISA specific to each molecule, as explained by Shinkaruk et al. [24]. The primary antibodies were selected and obtained in previous works [25,26]. All glassware was coated with silicone (Sigmacoat^®^, Sigma-Aldrich, Saint Quentin Fallavier, France, Ref SL2) and all IFs solutions were handled with low retention tips.

##### Characteristics of the ELISA

The soy-matter samples were diluted to 1/50 (*v*/*v*) for enzymatic digestion. The sensitivities of the GEN and DAI assays in food matter were 10 µg.mg^−1^ and 6.5 µg.mg^−1^, respectively. The intra-assay variation was never greater than 7% and the inter-assay variation obtained on different microtitration plates was always below 17% [25,26]. Assays were not considered if the “r” coefficient of the sigmoid calibration curve was <98.5%. The final dilutions in the microtitration plates ranged from 1/50 to 1/800. All values are given here in aglycone equivalent.

### 2.3. Statistical Treatments

All data correspond to a mean value and its standard error of the mean (SEM) derived from triplicate or quadruplicate measurements performed on three or four different microtitration plates. Due to the small number of values in each sample, a non-parametric statistical test, i.e., the Wilcoxon–Mann–Whitney test, was used. Sample values were sorted by rank, then the U parameter describing the intercalation of values of the two samples compared was obtained. The significance level (p) was determined using specific tables that provide this value according to the size of each sample. The final significance was half the value provided by the table for two-tailed tests. If the *p*-value was less than 0.05, it indicated that all the values of a triplicate or quadruplicate were lower than those of the other sample.

## 3. Results

Here, we present the IFs contents of tempeh and miso prepared according to traditional recipes. Data showing the impacts of water renewal during beans soaking on IFs contents for soy juice and tofu domestic preparations are given next. The impact of water rinsing on the IFs contents of textured proteins of different sizes is also shown. Finally, the results of rinsing tests of commercial tofu and tempeh are presented. The IFs levels in the okara samples collected during soy juices and tofu makings are presented in Supplementary Figure S1.

The concentrations of IFs were compared to those measured in commercial products bought in French supermarkets specifically for this study and also to those previously measured by our team for the same products using the same technique and published in [1]. The reference data issued in [1] are also presented in Table 1.

### 3.1. Impact of Traditional Process on Isoflavones Content in Tempeh and Miso

**Figure 1:**
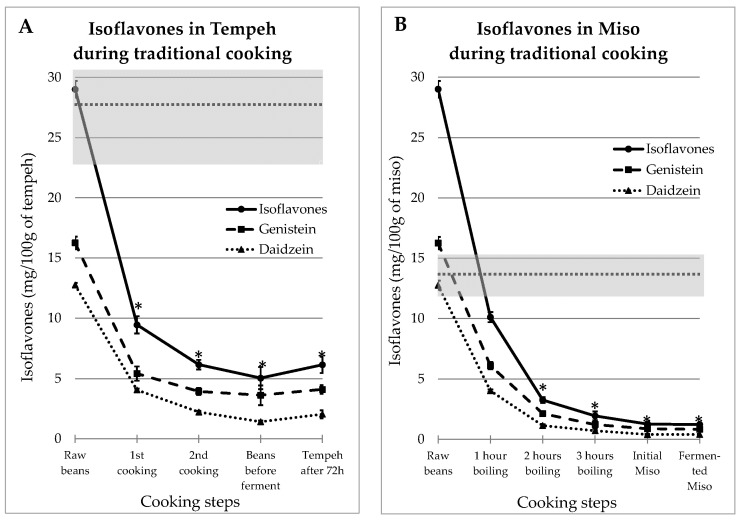
Evolutions of Genistein and Daidzein concentrations during the traditional preparations of tempeh (**A**) and miso (**B**). The gray zone covers the mean and SEM of the equivalent commercial products presented in Table 1. The stars indicate IFs values in food that were significantly different from the mean ± SEM of IFs content in commercial products (*p* < 0.05).

IFs levels measured at different stages of the traditional recipes of tempeh and miso are presented in Figure 1.

As mentioned above, tempeh and miso were both made from dehulled beans which contained 16.5 ± 0.52 mg GEN/100 g and 12.8 ± 0.17 mg DAI/100 g. The IFs concentrations measured at the different preparation steps decreased in an asymptotic way, as shown in Figure 1A for tempeh and Figure 1B for miso. The gray zones on the figure cover the range of values observed in industrial-equivalent food products. The stars indicate IFs values in prepared food that were significantly different from the mean ± SEM of IFs contents in commercial products (*p* < 0.05). It can be seen that in both cases, the resulting IFs levels were consistently much lower in “homemade” food than in commercial products. Moreover, this effect is the most important as soon as the first preparation step is complete; additional steps results in only slight reductions.

### 3.2. Impact of Water Renewals during Beans Soaking for Soy Juice and Tofu

#### 3.2.1. Process Tested on Whole Soybeans

**Figure 2:**
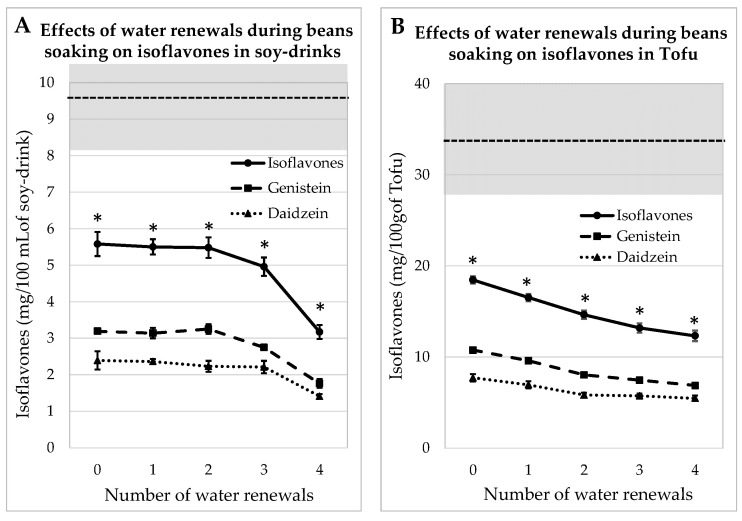
Evolutions of Genistein and Daidzein concentrations in soy juice (**A**) and in tofu (**B**) prepared from whole soybeans soaked for 12 h in water renewed several times (1 to 4) or not renewed (0). The gray zone covers the mean and SEM of the equivalent commercial products presented in Table 1. * indicates a significant difference from commercial products (*p* < 0.05).

IFs were assayed in soy juice and tofu prepared from whole beans, which contained 32.8 ± 0.9 mg GEN/100 g and 27.6 ± 0.12 mg DAI/100 g, soaked for 12 h with 0, 1, 2 or 3 water renewals. It should be noted that the two first soaking water batches contained dust and lipids, but the subsequent water batches were clear. The concentrations of aglycone IFs in mg/100 mL of soy juice and in mg/100 g of tofu are given in Figure 2. The amounts of IFs were always significantly lower (*p* < 0.05) than those measured in commercial equivalent products. After the fourth rinsing, the amount of IFs was reduced by 43% in the juice and by 33% in the tofu sample. Indeed, the envelope of the seed prevented IFs from leaking in the water during the soaking steps.

The IFs levels measured in the okara produced during soy juice and tofu manufacturing are shown in Supplementary Figure S1. It can be seen that the IFs decreased asymptotically. When okara was obtained from whole beans, 40% of IFs were removed after three water renewals, while almost 70% were eliminated after four water renewals on dehulled beans. The IFs decrease was significant after the first water renewal.

#### 3.2.2. Process Tested on Dehulled Beans

**Figure 3:**
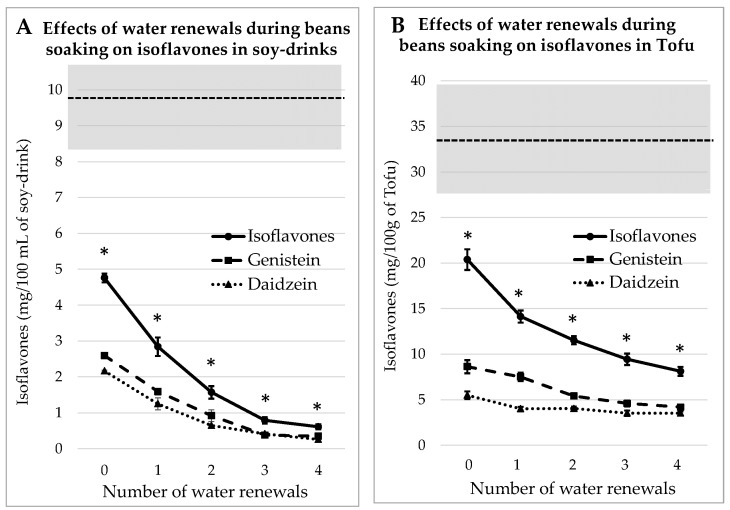
Evolutions of Genistein and Daidzein concentrations in soy juice (**A**) and in tofu (**B**) prepared from dehulled soybeans soaked for 12 h in water renewed 1 to 4 times or not renewed (0). The gray zone covers the mean and SEM of the equivalent commercial products presented in Table 1. * indicates a significant difference from commercial products (*p* < 0.05).

According to a traditional tofu-making experience in Debao Guangxi [11], it appears that the traditional recipe of tofu was based on dehulled beans. Therefore, the same soaking process as already described for whole beans was tested on dehulled beans. The concentrations of IFs, i.e., GEN + DAI, measured in each case are listed in Figure 3. As observed in the precedent case, the “homemade” juice and tofu always contain significantly (*p* < 0.05) less IFs than commercial equivalent products. When the number of water renewals increases, the IFs levels decrease in a quasi-asymptotic way.

It can also be inferred by comparison between Figure 3 and Figure 2 that the additional water renewals are more efficient at removing IFs when applied on dehulled beans than on whole beans. After four rinses, the IFs concentration in soy juice is reduced by 87%, while in tofu it was reduced by 60%. The reason is probably that the lack of a seed coat eases the IFs leakage into the water.

### 3.3. Impact of Water Renewals on the Levels of Isoflavones in Textured Proteins

The textured soy-proteins tested contained higher IFs concentrations than whole or dehulled soybeans. Indeed, although the different products did not come from the same soy matter, regular analysis of soybeans resulted in GEN + DAI concentrations of IFs aglycones around 60–70 mg/100g. In parallel, textured proteins were regularly measured at concentrations ranging from 90 to 110 mg/100g. This is because the matter used did not come from the same batches of beans. In addition, textured soy-proteins were obtained from soy defatted cakes resulting from soy seeds pressed for oil. As the extrusion process used did not significantly alter the IFs content and since oil represented about 20% of the mass of the initial seeds, the resulting textured soy-proteins were enriched in IFs. 

#### 3.3.1. Treatment with Water at 22 °C

**Figure 4:**
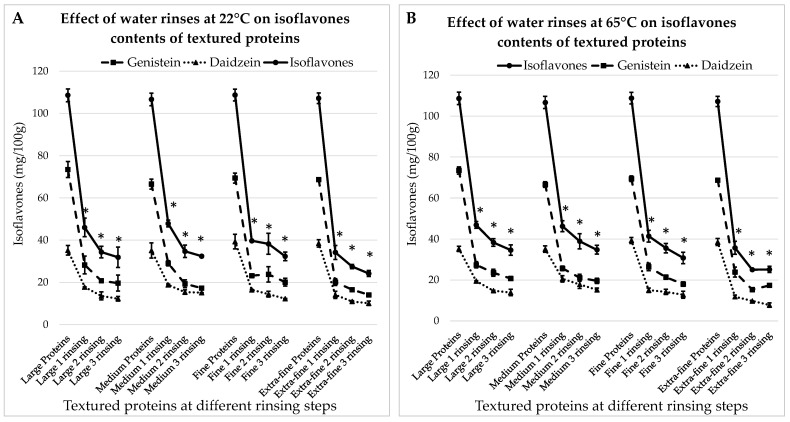
Effects of water rinses performed at 22 °C (**A**) and 65 °C (**B**) on the isoflavone contents of textured soy-proteins of different sizes, from large to extra-fine. * indicates a significant difference from initial IFs level (*p* < 0.05).

The concentrations of IFs in textured proteins hydrated for 30 min at room temperature with different rinsing steps are presented in Figure 4A. Here, the initial matter contained: 73.4 ± 3.8 mg GEN/100 g and 35.2 ± 2.3 mg DAI/100 g; 71.5 ± 2.4 mg GEN/100 g and 35.1 ± 3.6 mg DAI/100 g; 70.4 ± 2.4 mg GEN/100 g and 38.3 ± 3.5 mg DAI/100 g; 70.7 ± 0.8 mg GEN/100 g and 36.5 ± 1.7 mg DAI/100 g, for large, medium, fine and extra-fine particles, respectively.

From Figure 4A, it can be seen that in all cases, the first rinsing results in the greatest drop in IF levels (*p* < 0.05). After the third rinsing, the concentrations of IFs are decreased by 70.6%, 69.9%; 70.3% and 77.4% for large, medium, fine and extra-fine proteins, respectively. 

#### 3.3.2. Treatment with Water at 65 °C

The results for the treatment with hot water are presented in Figure 4B. They are very similar to the previous case: after the first rinsing, the concentrations of IFs decreased significantly (*p* < 0.05); after the third rinsing, they decreased by 68.1%, 67.4%, 71.7% and 76.5% for large, medium, fine and extra-fine particles, respectively. Hence, IFs’ removal efficiency is not significantly influenced by the soaking temperature. However, for both temperatures, the difference in the results is significant between the large and extra-fine proteins. At room temperature, after three rinsing steps, the total IFs contents are 31.9 ± 4.9 mg/100 g and 24.2 ± 1.5 mg/100 g in large and extra-fine textured proteins, respectively. At 65 °C, the results are 34.6 ± 2.6 mg/100 g and 25.1 ± 1.5 mg/100 g in large and extra-fine textured proteins, respectively.

### 3.4. Impact of Domestic Rinsing on Isoflavones Content of Commercial Tofu and Tempeh

**Figure 5:**
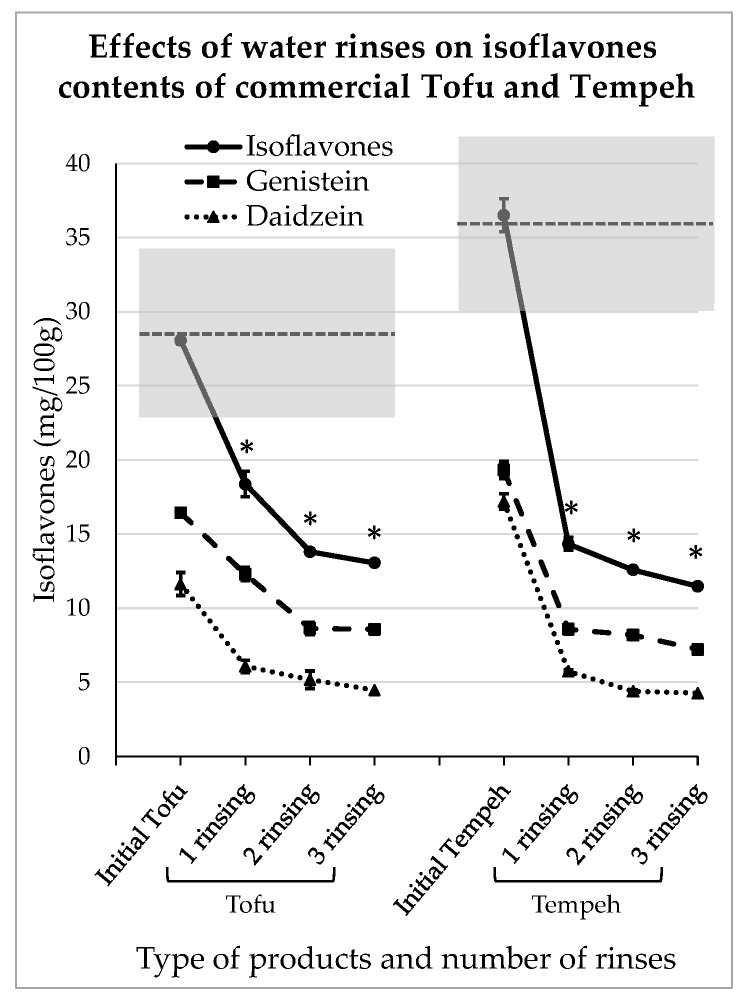
Reductions in isoflavones in commercial tofu and tempeh obtained after domestic water rinses. The stars indicate a significant difference in isoflavones concentration compared to the initial product (*p* < 0.05).

A consumer may prefer to buy ready-to-use tofu or tempeh, and of course in that case the levels of IFs can be quite high, as shown in Table 1. Of the commercial products used here for the domestic rinsing test, the plain tofu contained 16.4 ± 0.92 mg GEN/100 g and 11.6 ± 1.0 mg DAI/100 g and the plain tempeh contained 19.3 ± 0.18 mg GEN/100 g and 17.2 ± 0.44 mg DAI/100 g. The IFs levels corresponding to the different rinsing steps are presented in Figure 5.

Rinsing with water seems to be effective for commercial products provided that their texture is strong enough to withstand such treatment. In this experiment, the products were not significantly impregnated with water as they conserved their firmness and weight before and after each rinsing step. However, as can be seen in Figure 5, after just 10 min of contact with water, the IFs levels significantly decreased (*p* < 0.05). Indeed, in tofu, they reduced to 12.3 ± 0.17 mg and 6.1 ± 0.7 mg for GEN/100 g and DAI/100 g, respectively. After 20 min of contact and two water renewals, they were reduced to 8.57 ± 0.41 mg and 4.5 ± 0.6 mg of GEN/100 g and DAI/100 g, respectively. Similarly, in tempeh, after 10 min of rinsing, the IFs concentrations were reduced to 8.6 ± 0.6 mg and 5.7 ± 0.5 mg for GEN/100 g and DAI/100 g, respectively. At the end of the rinsing process, the amounts of IFs were reduced to 7.2 ± 0.03 mg and 4.3 ± 0.1 mg for GEN/100 g and DAI/100 g, respectively. As observed previously, the IFs removal followed an asymptotic scheme and the third rinsing step did not seem to be very efficient.

## 4. Discussion

Although raw soybean is the pulse with the highest content of proteins, it also contains phytic acid and tannins, which reduce mineral absorptions; oligosaccharides, which ferment in the gut, causing flatulencies; lipoxygenases, which decrease PUFAs quality; and saponins and sapogenins, which degrade the lipid membranes of enterocytes [27]. Soybeans also contain hemagglutinins, which can induce the coagulation of white and red blood cells if they enter the blood stream, and protease inhibitors, the Bowman–Birk and Kunitz factors, which reduce the proteins digestibility [27]. Finally, they also contain allergens and IFs [27]. This is why Baraibar Norberg and Deutsh [7] wrote that “*The same chemistry that protects the soybean against pests makes it partially indigestible for humans and conducive to flatulence and stomach pain*”. However, barely heating soybeans considerably reduces all antinutritional factors except IFs, which are thermoresistant. Fortunately, since IFs are in glyco-conjugated forms, they are soluble in water and may be reduced via water rinsing. This effect was tested in different contexts in this work.

### 4.1. Data Analysis

As seen in Table 1, industrial soy food currently found in supermarkets may contain quite high, although variable, amounts of IFs. Unfortunately, only very few have a mention of their IFs contents on the packaging. In France the Food Safety Agency advised in 2005 to limit IFs intake to 1 mg.kg^−1^day^−1^ [19]. As will be seen later, this “Reference Dose” should most probably be lowered nowadays due to toxicological results subsequently obtained by the USA NTP. The French Agency’s dose represents less than a portion of toasted soybeans for an adult of 60 kg. 

In this study, it also appears that the initial isoflavone levels in raw matters may vary. Indeed, the whole and dehulled soybeans used for this work were sourced from two different storage silos belonging to the same organic farmers’ union. As a result, they might have originated from different cultivation areas in the South-West of France. It is well known that culture conditions, including climate, soil quality and even farm management practices, may influence the amount of isoflavones in soybeans [28]. Therefore, such differences in raw materials were not unexpected. 

The experiments performed here show that IFs can easily be removed from soy matter via simple water treatments. This effect was already shown by several authors [29]. By implementing traditional recipes of tempeh and miso, in this work, it was possible to obtain foodstuffs containing almost 5 to 10 times less IFs than commercial equivalent products. This suggests that the ancient soy-consumption, traditionally in Asia, only provided small amounts of estrogenic IFs. This work also shows that simple water renewals during domestic beans soaking can dramatically reduce estrogenic IFs from subsequent preparations of soy juice or tofu. It appears that treating dehulled beans is more efficient for IFs removal than treating whole beans, as already observed [12]. Similarly, when water rinsing was tested on textured proteins of different sizes, IFs were more efficiently removed from fine or extra-fine proteins than from large or medium-sized ones. The difference in IFs levels between large and extra-fine proteins after water treatment was significant (*p* < 0.05). Moreover, using hot water did not bring additional efficiency to the process. These results are consistent with those previously published by Bensaada et al. [12]. Although this result might appear to be counterintuitive, it was observed several times by our team during the analysis of water treatments on both soybeans and soy-textured proteins. These observations suggest that in hot water, the proteins on the surface of a soy particle may coagulate faster than those from the center, thereby limiting isoflavones migration into the water. This hypothesis warrants further investigation using appropriate measurement techniques.

Regarding the proteins and lipids contents of soy products, they are not significantly reduced after water treatment [12]. Indeed, working on whole beans or dehulled beans allows the soy-cells integrity to be preserved. Thus, losses of large molecules are limited while small molecules such as IFs or vitamins and minerals can leak into the water when they are not adsorbed to larger biological cell constituents. In this study, vitamins and minerals were not analyzed based on the assumption that this issue should not constitute a major matter of concern, since this was already the case when soybeans were boiled and if the soy was a part of a diversified diet.

Finally, it may be difficult to perform these treatments at an industrial scale, and this is why the rinsing processes were also tested on commercial products and they proved to be efficient. Regardless, such water rinsing or soaking should be encouraged at domestic levels for most consumers who do not require estrogenic IFs intake. Food companies may also innovate, creating food products that could be easily treated at home.

### 4.2. Traditional Treatments

The ancient texts mentioning soybean and the actual soy-cooking practices, which derive from ancient traditions, both involve cooking with water. Such process was applied to reduce antinutritional factors, including IFs. This occurred when water was discarded. Soybean washing, soaking and boiling have been mandatory throughout the history of soy food [7]. In the past, boiling allowed to cook soybeans and to sterilize them before fermentation [9].

Considered as a low-value crop, soybean was initially used for animal feeding. However, under the Han dynasty, there was an increase in the Chinese population followed by a subsequent decline in livestock farming, and land was more often used to produce plant foods for humans instead. During this period, veganism developed in Asia and foods of animal origin were essentially based on fish, poultries and pork. During the territorial expansion wars of the Han period, soy became a war food, as it could be easily stored and transported. Because of its antinutritional factors, soy was not appreciated by human consumers and was always boiled. According to Lee et al. [9], fermentation was developed quite early to improve the taste and preservation of soy, and most probably to improve the nutritional quality of boiled soybeans. Indeed, the first remains of fermented soy were found with the first earthenware produced in Korea [9]. This production appeared to occur during the Bronze age. Tempeh, miso and natto fermentations allow the taste of soybeans to be improved and allow an increase in the vitamins of the group B [30]. This could compensate the losses of these water-soluble micronutrients during water treatments. Interestingly, Puri et al. [31] mentioned that soybean sterilization was mandatory prior to inoculation of soy matter and prior to fermentation. They reported that boiling considerably reduced IFs in subsequent fermented soy food. Sterilization via boiling was the process traditionally applied in ancient times. Water contact with seeds favored the leakage of glycosylated IFs into the cooking water. Nowadays, to save time and energy, sterilization processes are mainly performed by steaming under pressure using an autoclave [30,32]. However, it appears that steaming reduces the contact between soy matter and water maintaining larger amounts of IFs in final foodstuffs [33]. Consequently, the estrogenic IFs levels are higher in steamed food than in boiled food. Additionally, while this effect may be beneficial when IFs are required and useful, it constitutes a risk when they reach excessive levels.

### 4.3. Health Effects

#### 4.3.1. Traditional Pharmacopeia

Soy’s polyphenols content has been studied thoroughly. Thus, soybean is known to contain many functional substances such as phenolic acids, flavonoids, IFs, stilbenes, saponins [30], or the peptide lunasin. According to Baraibar-Norberg and Deutsh [7] soybean first appeared in the traditional Chinese pharmacopeia. The oldest treatise reporting soy medicinal properties that is still available is the *Shiliao bencao* (*Compendium of Diet Therapy [Materia Dietetica]*), which was written by Meng Shen in 670 CE. It states that “*soybeans boiled into a liquid form, can eradicate poisons from the system and cure gastric fever, paralysis, pains, difficulty in passage of urine and other bladder troubles. It can also improve circulation of the blood, improper heart, liver, kidneys and stomach function and even remedy the chills”* [7,34]. It should be noted that none of these health effects rely on estrogenic properties. This observation suggests that IFs were not present in these preparations, confirming that traditional processes tended to eliminate them. By comparison, kudzu roots, which contain GEN, DAI and puerarin, among other substances, were known to induce some estrogenic and antipyretic effects. The latter are now attributed to IFs [35] and suggest that the traditional preparation of kudzu preserved GEN and DAI levels [35]. These facts put together with data obtained on IFs content of soy foods prepared following traditional recipes, strongly suggest that the exposure to IFs was low in the pre-industrialized period. It probably increased in human populations with the advent of industrial processing.

#### 4.3.2. Toxic Effects and Reference Doses

The reprotoxic effects of soy IFs and their methoxylated parents from clover were first documented in the 1940s when ewes were affected by the clover disease [36]. Within four years, the herds’ fertility reduced from 80% to less than 5% and many farmers stopped their activities. Veterinarians then came to understand that IFs reduced the pituitary hormones FSH and LH, which themselves were decreased due to an impairment of GnRH production at hypothalamic level [37]. Such a mechanism is known to be used pharmacologically in contraceptive pills based on synthetic estrogen: ethynyl-estradiol [38]. This impairment of pituitary hormones was then observed in premenopausal women who had 45 mg dietary IFs per day [39]. More recently, it was shown that exposure to 50 mg IFs per day significantly increases the risk of luteal deficiency [40] and infertility [41] in American women. An exposure of baby girls to soy IFs via soy-based infant formulas was shown to increase the incidence of fibrosis, endometriosis, menstrual pains and bleedings [42,43,44,45]. These impairments were also observed in women who engaged in overconsumption of soy-based products [46]. Moreover, a case of hypogonadism, gynecomastia and reduced libido was observed in a Japanese man who overconsumed soymilk for four years [47]. Finally, in five studies, soy IFs in biological fluids were associated with reduced quality and quantity of semen in men [48,49,50,51,52].

In addition, a controversy remains regarding the effects of IFs on breast cancers. An obvious discrepancy was observed between two data sources. On one hand, there are data obtained in vitro on recognized cell models [3,53], in animals implanted with these cancer cells [3,54], in healthy premenopausal women under IFs oral supplementation [3,55] and in women with breast cancer supplemented with soy and IFs [3,56]. On the other hand, there are epidemiological studies [3,57] and reviews of randomized controlled trials (RCTs) [3,58]. The population studies tend to show a reduction in breast cancer risk in Asian women in an Asian environment (including green tea polyphenols intake) but this protection has not been clearly observed in Western women in a Western environment [3,59]. Many biases, including genetic, dietary and environmental parameters, make it difficult to reach a definitive conclusion on the effects of IFs on breast cancer incidence in Western menopausal women. The reviews including RCTs concluded that there was no increased risk of breast cancer in women taking soy or food supplements based on soy IFs [3,60]. However, the subjects recruited for these RCTs were selected based on health criteria and did not represent the real population. Moreover, the RCTs were not designed to study the incidence of breast cancers, and thus the conclusions of these analyses should be considered cautiously.

Still, there is now sufficient evidence to indicate that soy IFs can interact with human thyroid function. On top of mechanistic arguments [3], clinical cases [61,62], observation studies [63,64] and intervention studies [65] confirm that soy IFs have slight anti-thyroid effects and can worsen the status of hypothyroid patients.

Beside the effects directly reported in human beings, the USA NTP showed that GEN was reprotoxic in Sprague Dawley rats in a multi-generational study published in 2008 [4]. The study showed that some reproductive parameters, including anogenital distance in pups and litter size, were altered at a GEN dose of 35 mg/kg/day in males. According to the general toxicology rules, the NTP study can be used to define a Reference Dose in humans by applying safety factors to the level defined. Indeed, an amount of 35 mg.kg^−1^day^−1^ was the LOAEL (Lowest Observed Adverse Effect Level) and no effects were recorded on reproductive parameters at a dose of 7 mg.kg^−1^.day^−1^. Usually, when a LOAEL is available, safety factors are required, including 10 for interspecific differences between rats and humans, 10 for intraspecific differences, i.e., interindividual differences between human beings; and 1.8 or 3 for conversion of LOAEL to NOAEL. If the last factor is fixed at 1.8, the Reference Dose for GEN appears to be about 20 mg.day^−1^ for an adult weighing 60 kg. In addition, as GEN is usually present in food with DAI, both IFs should be considered. Unfortunately, there is neither LOAEL nor NOAEL for DAI in rats. Additionally, even if DAI is less estrogenic than GEN, it can be converted into Equol, which may be more active than GEN on certain tissues [3]. Consequently, it would be relevant to consider the limit concentration of both GEN and DAI to be set at 20 mg.day^−1^ for an adult weighing 60 kg. The present study showed that the IFs levels reached in tofu, tempeh, miso and soy juice were below this limit after water treatments. Meanwhile, this was not the case in the corresponding industrial products. For textured proteins, the treatments allowed the IFs concentrations to decrease at levels from 35 to 22 mg/100 g depending on protein sizes. This is higher than the potential Reference Dose. However, textured proteins are usually used with other ingredients and at a percentage ranging from 45 to 20%. Therefore, the final concentration in a 100g portion is lower than 20 mg. This dose is about half that which was shown to have a physiological effect in humans and seems to correspond to exposure levels in Asian populations following traditional soy food preparation processes. Finally, the various doses discussed here correspond to median modern exposures in China and Japan [15,16]. To our knowledge, they did not induce deleterious effects in the past.

To conclude this part, excess IFs should be avoided by populations which should not be exposed to estrogens. These include infants, children, premenopausal women, pregnant women, hypothyroid patients and men. Concerning women with breast cancer, it seems difficult to advise on soy consumption, considering the estrogenic effects of IFs. The latter may prevent breast cancer occurrence, but their actions on an established breast cancer dependent on estrogens are still a subject of debate.

#### 4.3.3. Beneficial Health Effects for a Restricted Population

If IFs should be carefully monitored, it is because they seem to be active in human beings at dietary levels. Their beneficial estrogenic effects are restricted to certain categories of populations. Indeed, the most studied effect is the reduction in menopausal symptoms. The last meta-analysis which analyzed the effects of IFs on vasomotor symptoms and criteria showed only modest effects [66]. However, other meta-analyses reported a decrease in the occurrence of hot flushes [67]. In some studies, the effects appeared to be restricted to some natural compounds such as Equol [68], while in others, only some preparation types were found to be active [69]. Nevertheless, while the latest studies tend to show that vasomotor symptoms equally affect Asian and Western peri- and post-menopausal women [70] in accordance with an Asian exposure to IFs lower than 50 mg/day, IFs are the most popular substances used worldwide to reduce hot flushes.

Besides menopausal symptoms, there is evidence that IFs doses >80 mg /day can prevent bone mineral density (BMD) decrease in menopausal women [71]. Equol is also considered to have a specific beneficial effect on BMD during menopause when administered as supplement [72]. In the latter study, an RCT gathered 76 menopausal women between 50 and 55 years old. The treatment lasted one year and the supplement contained 80 mg IFs aglycone, 10 mg of Equol and 25 mg Resveratrol as an antioxidant. In these specific conditions, the BMD was preserved significantly in the treated group compared to the placebo group.

To conclude, there are sufficient data showing that IFs may be useful for menopausal women showing no signs of thyroid or estrogen-dependent diseases.

### 4.4. Consequences of this Finding on the Estimation of the Population’s Exposure

This study has shown that simple water treatments like water rinsing, soaking or boiling can dramatically reduce the content of IFs in soy foods. It also gathered historical testimonies indicating that such water treatments were traditional when preparing soy food in Asia, as soybeans were eaten increasingly often. The historical data show that fermentation was developed quite early as a technique to improve the digestibility, taste and preservation of soy. Looking at actual recipes reported by Asian consumers, it appears that homemade soy is generally processed following family recipes, and thus using water treatments. However, nowadays, traditional boiling in water tends to be replaced by steaming, which reduces water-to-beans contact and maintains the IFs in soy foods. 

Hence, the most significant result of this work is the large differences observed between water-treated products and those found on the market due to the limited water treatments performed in the industry to reduce energy and environmental costs. Thus, the importance of checking the origin of soy foods when estimating IFs intakes is highlighted. This is particularly true in Asia, where a larger percentage of people may be prompted to prepare soy at home according to family recipes. Table 2 below shows the discrepancies between IFs plasma levels, as found in different studies where IFs intakes were estimated.

As can be seen in Table 2, the plasma IFs levels are much higher in Western people under an equivalent estimation of IFs exposure. The latter were generally assessed by assaying commercial soy foods found in the corresponding local markets. Of course, at least part of the differences may come from analytical techniques and the blood sampling time. However, in Asia, if IFs are ingested on a regular basis, e.g., every day, a steady-state level may be expected. Therefore, whatever the time of sampling, the blood levels should be fairly stable. On the other hand, in Western countries, where soy intake remains irregular, the time of sampling plays a major role on blood IFs concentrations, and this may explain the low blood levels recorded in [78]. In addition, in [14], it was shown that there are no fundamental differences in IFs metabolism and pharmacokinetics between Asian and Western consumers. Thus, the intake estimation should be assessed to explain the discrepancies appearing in Table 2.

While it is likely that in the West, soy food is mainly obtained from industrial sources, as there is no tradition of soy cooking, in Asia, it is customary to prepare soy at home according to family recipes. Therefore, the intake estimation may be over-evaluated in Asia if it is only based on industrial products, as described in the works of Kimira et al. [75] and Chan et al. [82]. This comment should be considered while discussing a Reference Dose for soy IFs.

## 5. Conclusions

In population studies, the distinction between domestically and industrially processed soy foods has not been made, potentially leading to an overestimation of soy consumers’ overall exposure to IFs. This is particularly true in Asian contexts, where traditional soy-cooking practices prevail. A comprehensive review of traditional soy-processing methods revealed a consistent practice: soybeans were invariably boiled, either as a precursor to subsequent fermentation steps or to enhance their digestibility. This study shows that traditional and domestic processing methods significantly reduce the content of IFs in soy-based foods compared to industrial methods. The application of two or three water treatments aids in achieving the potential “Reference Dose” for IFs, established according to the toxicological studies of the US National Toxicology Program. Furthermore, the results corroborate previous findings that water treatments can significantly decrease IFs in soy foods.

Given the functional activities of IFs, their use should be judicious. One approach to achieve this goal is by controlling dietary exposure levels through the introduction of water treatments at both industrial and domestic soy food preparation stages, thereby ensuring a safer democratization of their consumption. This simple rule could indeed result in a substantial shift in the global human exposure to estrogenic substances. 

## Figures and Tables

**Table 1:** Genistein and daidzein amounts per portion of different types of soy food collected on the French market.

Type of Product	Number of Measurements	Portion Size (g)	Mean GEN + DAI/ Portion (mg)	Standard Error of Mean/Portion	Range of Measurements *
Raw soybeans	5	100	84.6	8.3	72.5 to 100.6
Toasted soybeans	5	100	134.0	37.9	61.9 to 247.7
Edamame	2	120	49.2	14.1	35.1 to 63.3
Soybean flour	2	100	38.5	5.5	32.9 to 43.9
Protein isolate (sports)	3	165	52.2	12.3	39.8 to 64.5
Soy-based drinks	9	100	9.9	1.7	2.8 to 17.7
Soy-based dessert cream	11	150	12.3	2.8	2.5 to 33.4
Soy-based yogurt	14	125	15.9	3.8	5.1 to 31.5
Soy-based cream	12	50 mL	4.5	5.9	3.1 to 6.7
Soy-based Vegan steak	19	100	27.8	3.5	4.1 to 51.2
Soy sausages	4	90	24.8	7.3	11.1 to 44.1
Soy-based raw pasta	2	100	23.7	1.6	22.1 to 25.3
Soy-based cooked pasta	2	100	8.75	0.6	8.2 to 9.3
Soy-based infant formulas	6	4-month-old infants	25.2	2.9	15.7 to 34.3
Tempeh	4	100	27.8	4.5	15.5 to 34.3
Tofu	13	100	34.8	5.9	9.4 to 79.8
Soy-based cheese	8	50	23.8	7.4	2.9 to 36.2
Miso	2	100	14.7	0.98	13.7 to 15.6
Soy sauce	9	10 mL	0.16	0.004	0.16 to 0.17

* Figures calculated from [1] and isoflavones are genistein + daidzein concentrations in aglycone forms.

**Table 2:** Review of isoflavones concentrations in human plasma in relation to estimated isoflavone intakes.

Location	Estimation Method	Estimated IFs Intake (mg)	IFs Plasma (nM)	Time of Plasma Collection	Study
Los Angeles	Hawaii Food Composition Database based on commercial items	23.72	30.1	No specific instruction	[73]
China (Shanghai)	USDA-ISU database based on commercial items	23.5	106.3	No specific instruction	[74]
		58.15	119.9		
		84.5	146.3		
		284.0	188.3		
Japan	From [75] (commercial items)	46.4	419.2	Overnight fasting	[76]
Japan	From [75] (commercial items)	40.8	411.0	After fasting > 5h	[77]
England	USDA-ISU database based on commercial items	33.7	510	Not precise	[78]
Japan	From [75] (commercial items)	32.5	553 *	No specific Instruction	[79]
		34.8	605 *		
The Netherlands	Strictly controlled soy-protein diet	48.5	2160	Fasting samples after chronic intake	[80]
		100.1	2530		
		104.2	2900		
		93.9	4800		
Hawaii	USDA-ISU database based on commercial items	96.0	5600	12h fasting and chronic intake	[81]

* Figures calculated from values expressed in ng.mL^−1^ from [79].

## Data Availability

The original contributions presented in the study are included in the article, further inquiries can be directed to the corresponding author.

## Supplementary Materials

The following supporting information can be downloaded at https://www.mdpi.com/article/10.3390/foods13070999/s1. Figure S1. Effect of water renewals during beans soaking on the level of isoflavones in Okara. A. Okara made from entire beans. B. Okara made from dehulled beans. The stars indicate a significant difference with 0 renewal (*p* < 0.05)
