# Supplementary material for: Traditional and Domestic Cooking Dramatically Reduce Estrogenic Isoflavones in Soy Foods
Source: Foods. 2024 Mar 25;13(7):999. doi: 10.3390/foods13070999 (PMC11011382; doi:10.3390/foods13070999)
Supplement: Supplementary file 1 [file foods-13-00999-s001.zip › foods-2931588-supplementary.pdf]

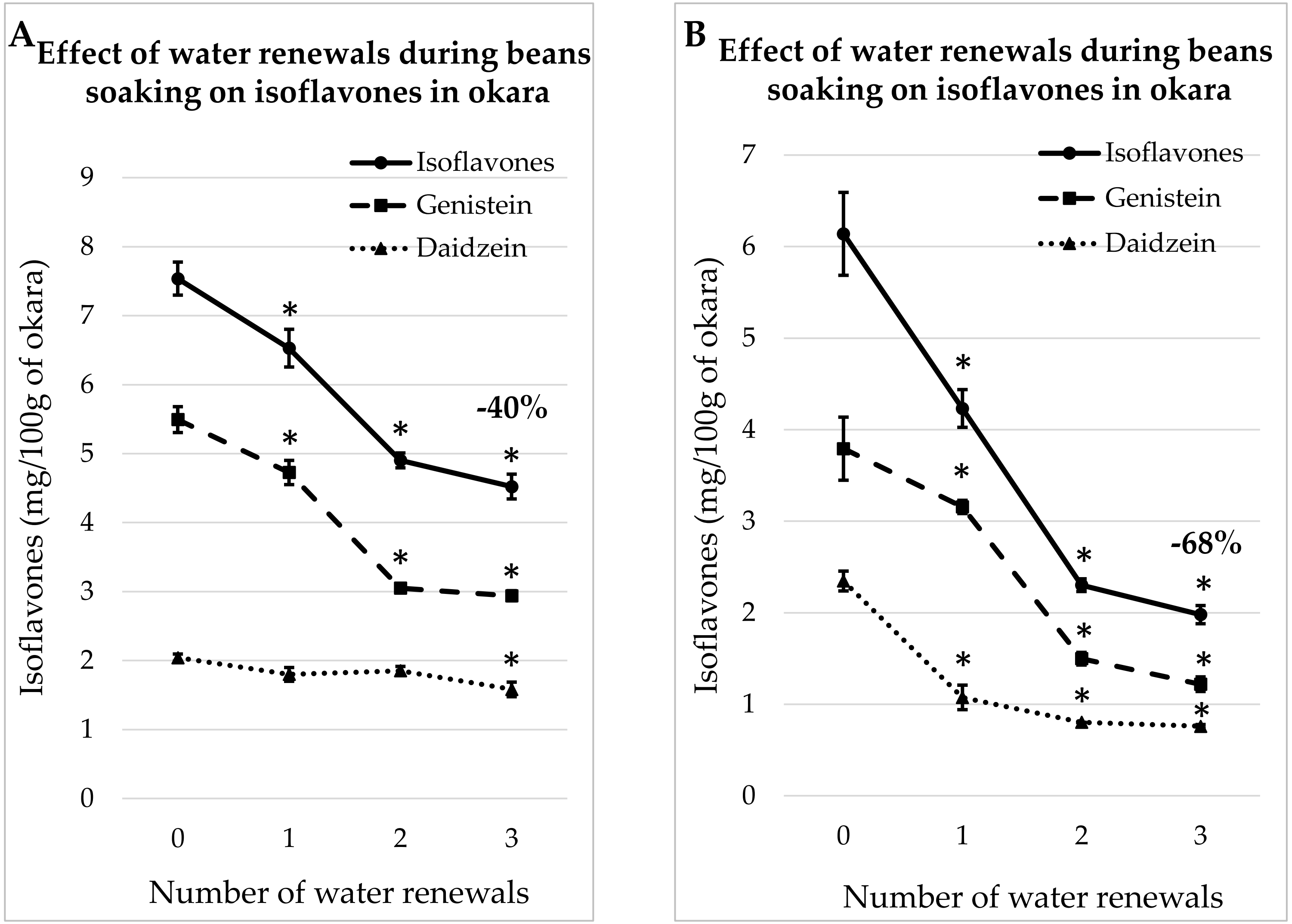

Supplementary figure S1. Effect of water renewals during beans soaking on the level of isoflavones in Okara. **A**. Okara made from entire beans. **B**. Okara made from dehulled beans.
The stars indicate a significant difference with 0 renewal (p<0,05)
